# A Meta-Analysis of the Genome-Wide Association Studies on Two Genetically Correlated Phenotypes Suggests Four New Risk Loci for Headaches

**DOI:** 10.1007/s43657-022-00078-7

**Published:** 2022-11-18

**Authors:** Weihua Meng, Parminder S. Reel, Charvi Nangia, Aravind Lathika Rajendrakumar, Harry L. Hebert, Qian Guo, Mark J. Adams, Hua Zheng, Zen Haut Lu, 23andMe Research Team, Debashree Ray, Lesley A. Colvin, Colin N. A. Palmer, Andrew M. McIntosh, Blair H. Smith

**Affiliations:** 1 Nottingham Ningbo China Beacons of Excellence Research and Innovation Institute, University of Nottingham Ningbo China, Ningbo, 315100 China; 2 Division of Population Health and Genomics, Ninewells Hospital and Medical School, University of Dundee, Dundee, DD2 4BF UK; 3 Division of Psychiatry, Edinburgh Medical School, University of Edinburgh, Edinburgh, EH10 5HF UK; 4 Department of Anaesthesiology, Tongji Hospital, Tongji Medical College, Huazhong University of Science and Technology, Wuhan, 430030 China; 5 PAPRSB Institute of Health Sciences, Universiti Brunei Darussalam, Bandar Seri Begawan, BE1410 Brunei Darussalam; 6 23andMe, Inc., Sunnyvale, CA 94086 USA; 7 Department of Epidemiology, Bloomberg School of Public Health, Johns Hopkins University, Baltimore, MD 21205 USA; 8 Department of Biostatistics, Bloomberg School of Public Health, Johns Hopkins University, Baltimore, MD 21205 USA

**Keywords:** Headache, Migraine, Unified Score-based Association Test, Correlated phenotypes, Meta-analysis, Genome-wide association study

## Abstract

Headache is one of the commonest complaints that doctors need to address in clinical settings. The genetic mechanisms of different types of headache are not well understood while it has been suggested that self-reported headache and self-reported migraine were genetically correlated. In this study, we performed a meta-analysis of genome-wide association studies (GWAS) on the self-reported headache phenotype from the UK Biobank and the self-reported migraine phenotype from the 23andMe using the Unified Score-based Association Test (metaUSAT) software for genetically correlated phenotypes (*N* = 397,385). We identified 38 loci for headaches, of which 34 loci have been reported before and four loci were newly suggested. The *LDL receptor related protein 1* (*LRP1*)—*Signal Transducer and Activator of Transcription 6* (*STAT6*)—*Short chain* *Dehydrogenase*/*Reductase family 9C member 7* (*SDR9C7*) region in chromosome 12 was the most significantly associated locus with a leading *p* value of 1.24 × 10^–62^ of rs11172113. The *One Cut homeobox 2* (*ONECUT2*) gene locus in chromosome 18 was the strongest signal among the four new loci with a *p* value of 1.29 × 10^–9^ of rs673939. Our study demonstrated that the genetically correlated phenotypes of self-reported headache and self-reported migraine can be meta-analysed together in theory and in practice to boost study power to identify more variants for headaches. This study has paved way for a large GWAS meta-analysis involving cohorts of different while genetically correlated headache phenotypes.

**Supplementary Information:**

The online version contains supplementary material available at 10.1007/s43657-022-00078-7.

## Introduction

Headache is one of the commonest symptoms that present to clinicians in general practice or in specialist neurology clinics (Fuller and Kaye 2007). Its lifetime prevalence in individuals is as high as 93% (Boardman et al. 2003). Globally, around 46% of the adult population suffers with an active headache disorder (Stovner et al. 2007). According to the current definitions of the International Headache Society, headaches can be classified into three categories: (1) primary headaches (including migraine, tension-type headache, and trigeminal autonomic cephalalgias); (2) secondary headaches (including headaches attributed to other disorders such as trauma, infection); (3) painful cranial neuropathies, other facial pain and other headaches (Headache Classification Committee of the International Headache Society 2018).

Among all types of headache, tension-type headache is the commonest form, causing over 40% of all headaches in the general population, while migraine is the most disabling type at population level, with a prevalence of around 10% of all headaches (Riesco et al. 2017). It is important to note that an individual can experience more than one type of headache at the same time (Fuller and Kaye 2007).

According to the Global Burden of Diseases 2019 study, headache disorders represent the 14th leading cause of disability-adjusted life years (DALYs) when considering all ages and both genders in 174 causes (GBD 2019 Diseases and Injuries Collaborators 2020). It was estimated in 2003 that migraine alone costs the UK over two billion pounds a year (Steiner et al. 2003). Migraine is still one of the leading causes of disability among over 300 diseases (Steiner et al. 2020).

It has been confirmed that headaches such as migraine are heritable. The single nucleotide polymorphism (SNP) based-heritabilities of migraine and self-reported headache were 0.15 and 0.21 in Caucasians, respectively (Gormley et al. 2016; Meng et al. 2018). Genome-wide association studies (GWAS) have revealed that there are significant genetic components contributing to migraine (Anttila et al. 2010, 2013; Chasman et al. 2011; Freilinger et al. 2012; Ligthart et al. 2011). A GWAS meta-analysis paper consisting of 22 cohorts by Gormley et al. identified 38 genetic loci for migraine (Gormley et al. 2016). Our study based on the UK Biobank resource also revealed 28 risk loci for self-reported headache, of which 14 loci had been previously identified by Gormley et al. (2016) and 14 loci were newly reported (Meng et al. 2018). A recent large GWAS meta-analysis on migraine has suggested 123 migraine-related loci (Hautakangas et al. 2022).

Recently, researchers have been encouraged to perform GWAS meta-analysis on genetically correlated phenotypes, due to the increasing recognition of pleiotropy in GWAS, to boost study power to detect more genetic components (Masotti et al. 2019). Pleiotropy refers to the phenomenon where a genetic variant or a gene has non-zero effect on multiple phenotypic traits and can contribute to genetic correlations among these traits (Stearns 2010). Successful examples of recent GWAS meta-analysis studies on genetically correlated phenotypes have included education and intelligence as well as different hypertension phenotypes (Hill et al. 2019; Zhu et al. 2015).

In a previous study, we reported that the self-reported headache phenotype from the UK Biobank and the self-reported migraine phenotype from the 23andMe were genetically correlated, with a high correlation value of 0.72 (*p* = 1.66 × 10^–68^, standard error = 0.04) (Meng et al. 2020). Therefore, we aimed to perform a joint GWAS meta-analysis study of these two different but highly genetically correlated phenotypes with a view to replicating previously identified genetic associations and identifying new associations arising from the increased power of this approach.

## Materials and Methods

### Cohorts’ Information

The two sets of GWAS summary statistics used in this study were from the GWAS on self-reported headache based on the UK Biobank cohort and the GWAS on self-reported migraine provided by the 23andMe (Gormley et al. 2016; Meng et al. 2018).

The definitions of self-reported headache (UK Biobank) were as follows: cases (*N* = 74,461), defined as those who self-reported headache symptoms affecting daily lives within last month using the UK Biobank online questionnaire; controls (*N* = 149,312), defined as those who did not have any pain affecting daily lives within last month (UK Biobank code 6159). The corresponding GWAS analysis was performed using a linear mixed model adjusting for age, sex, nine population principal components, genotyping arrays, and assessment centers (Meng et al. 2018). The dataset contains 9,304,965 SNPs (minor allele frequency > 0.005, imputation score > 0.1).

The definitions of self-reported migraine (23andMe) were: cases (*N* = 30,465), defined as those who self-reported a migraine history (diagnosed by doctors or self-diagnosing) using the 23andMe online questionnaire; controls (*N* = 143,147), those who self-reported having no migraine. The corresponding GWAS was performed using a linear mixed model adjusting for age, sex, and five population principal components (Gormley et al. 2016). The dataset contains 19,023,436 SNPs (containing minor allele frequency and imputation score information for all SNPs). This dataset was also used by Gormley et al. (2016).

All of the participants in both GWAS were of European descent. In addition, as the UK Biobank cohort only recruited within the UK, while the 23andMe mainly recruited from the USA, there was little sample overlap between the two cohorts (linkage disequilibrium score regression intercept = 0.009) (Meng et al. 2020). The detailed cohorts’ information and the statistical methods of the two GWAS can be found in the original papers (Gormley et al. 2016; Meng et al. 2018).

### The Preprocessing of the GWAS Summary Statistics

SNPs in both datasets were coded in a forward direction and according to the GRCh37 genome build. In total, 8,500,802 SNPs with minor allele frequency > 0.005 in both datasets were extracted. To ensure the datasets could be jointly meta-analysed, these SNPs were checked for same effect alleles and flipped accordingly in R (https://www.r-project.org/).

### The Meta-Analysis Method

The Unified Score-based Association Test (metaUSAT) is a software package for performing GWAS meta-analysis studies on genetically correlated phenotypes (Ray and Boehnke 2018) (https://github.com/RayDebashree/metaUSAT). The metaUSAT software applies a multivariate meta-analysis approach instead of a univariate approach of analysing each related trait separately. Unlike traditional GWAS meta-analysis on a single trait, where several sets of summary statistics on a single trait are combined into a single summary measure for that trait, the multivariate meta-analysis implemented by metaUSAT does not combine the summary statistics; instead, a joint analysis is performed using summary statistics from related traits. It is a statistical inference approach that leverages related traits to provide a *p* value for the test of no association of any trait with a SNP against the alternative that at least one trait is associated with the SNP. Being a complex data-adaptive approach, the metaUSAT software does not output an overall effect size (Beta) and standard error (SE) values for each SNP. The metaUSAT software is robust to the association structure of correlated traits and potential sample overlap (Ray and Boehnke 2018).

### The Annotation Method

The output generated from metaUSAT was uploaded to Functional Mapping and Annotation (FUMA v1.3.6b) for SNP annotation (Watanabe et al. 2017). We used the 1000 Genome Phase 3 reference panel by default and other default values adapted by FUMA in terms of defining lead SNPs and risk loci. FUMA also generates a Manhattan plot and a corresponding Q-Q plot for the meta-analysis result (https://fuma.ctglab.nl/). FUMA uses “maximum distance of linkage disequilibrium (LD) blocks to merge’’ (default value = 250 kb) to determine the number of associated loci and the* r*^2^ value (default value *r*^2^ ≥ 0.6 to be considered as non-independent) to determine the number of independent significant SNPs. In addition, the gene-based association analysis and the gene-set analysis were performed with Multi-marker Analysis of Genomic Annotation (MAGMA v1.08), which was integrated in FUMA (de Leeuw et al. 2015). In gene-based association analysis, summary statistics of SNPs were aggregated to the level of whole genes, testing the joint association of all SNPs in the gene with the phenotype. In other words, all the SNPs were mapped to 19,436 protein coding genes if the SNPs are located within genes. In gene-set analysis, individual genes were aggregated to groups of genes sharing certain biological, functional or other characteristics. This was done to provide insight into the involvement of specific biological pathways or cellular functions in the genetic aetiology of a phenotype. A total of 10,894 gene sets were tested and a competitive test model was applied. Tissue expression analysis was obtained from the Genotype-Tissue Expression (GTEx) project (https://www.gtexportal.org/home/) which was also integrated in FUMA. In the tissue expression analysis, average gene-expression per tissue type was used as gene covariate to test positive relationships between gene expression in a specific tissue type and genetic associations. In addition, regional plots of the suggested new loci were generated by LozusZoom (http://locuszoom.org/).

## Results

**Fig. 1:**
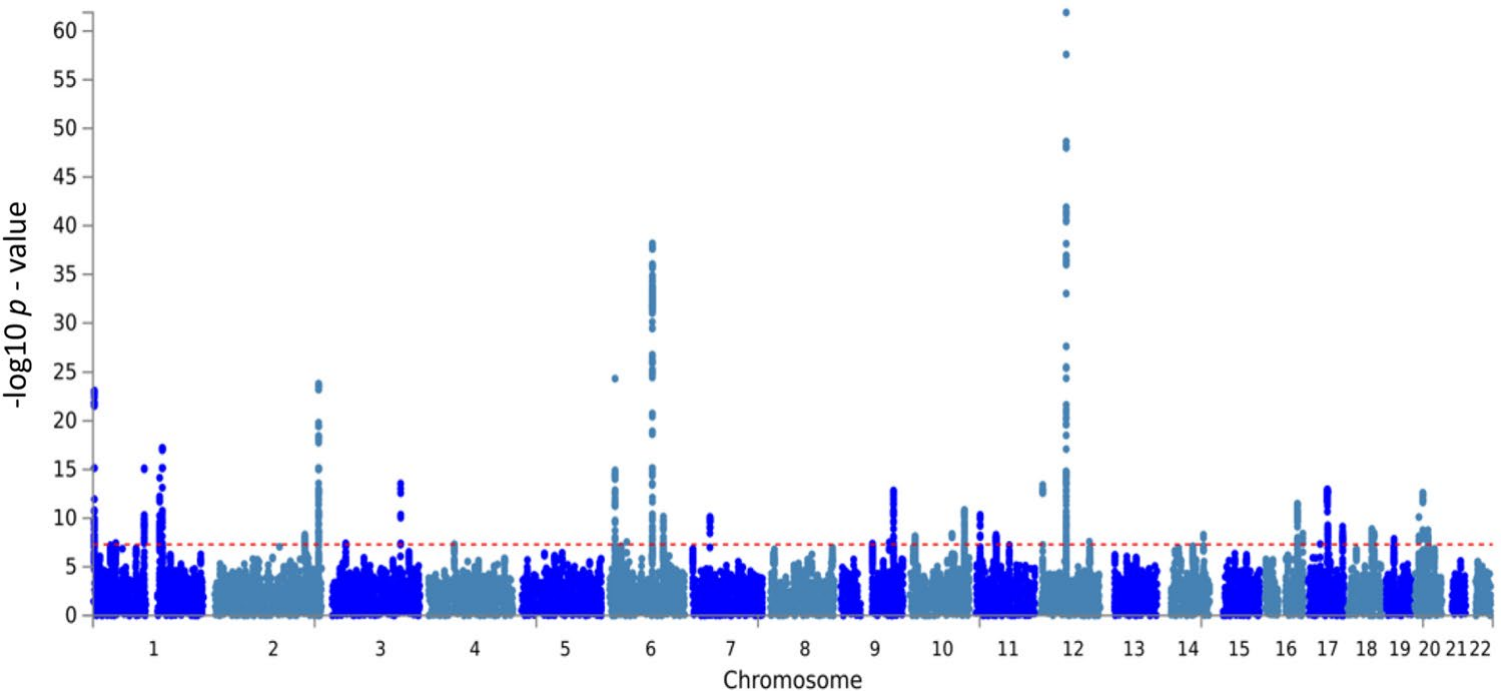
The Manhattan plot of the GWAS meta-analysis on headaches (*N* = 397,385). The dashed red line indicates the cut-off *p* value of 5 × 10^−8^

There were 8,500,802 common SNPs from both cohorts analysed by the metaUSAT software. FUMA reported 38 independent genetic loci across autosomal chromosomes with the *LDL* *Receptor related Protein 1* (*LRP1*)—*Signal Transducer and Activator of Transcription 6* (*STAT6*)—*Short chain Dehydrogenase*/*Reductase family 9C member 7* (*SDR9C7*) region in chromosome 12q13.3 being the most significantly associated locus with a leading *p* value of 1.24 × 10^–62^ for rs11172113. The Four And A Half LIM Domains 5 (*FHL5*)—UFM1 Specific Ligase 1 (*UFL1*) locus in chromosome 6q16.1 was the second most significantly associated, with a *p* value of 6.57 × 10^–39^ for rs9486715. A Manhattan plot showing these loci is shown in Fig. 1. A corresponding Q-Q plot is included as shown in Supplementary Fig. 1. Among the 38 identified loci, there were 2228 SNPs that demonstrated an association with genome-wide significance, with *p* value < 5 × 10^–8^. Among these SNPs, 113 SNPs were considered as independent associations (*r*^2^ < 0.6 with any SNP within the 2228 SNPs) (Supplementary Tables 1 and 2).

**Fig. 2:**
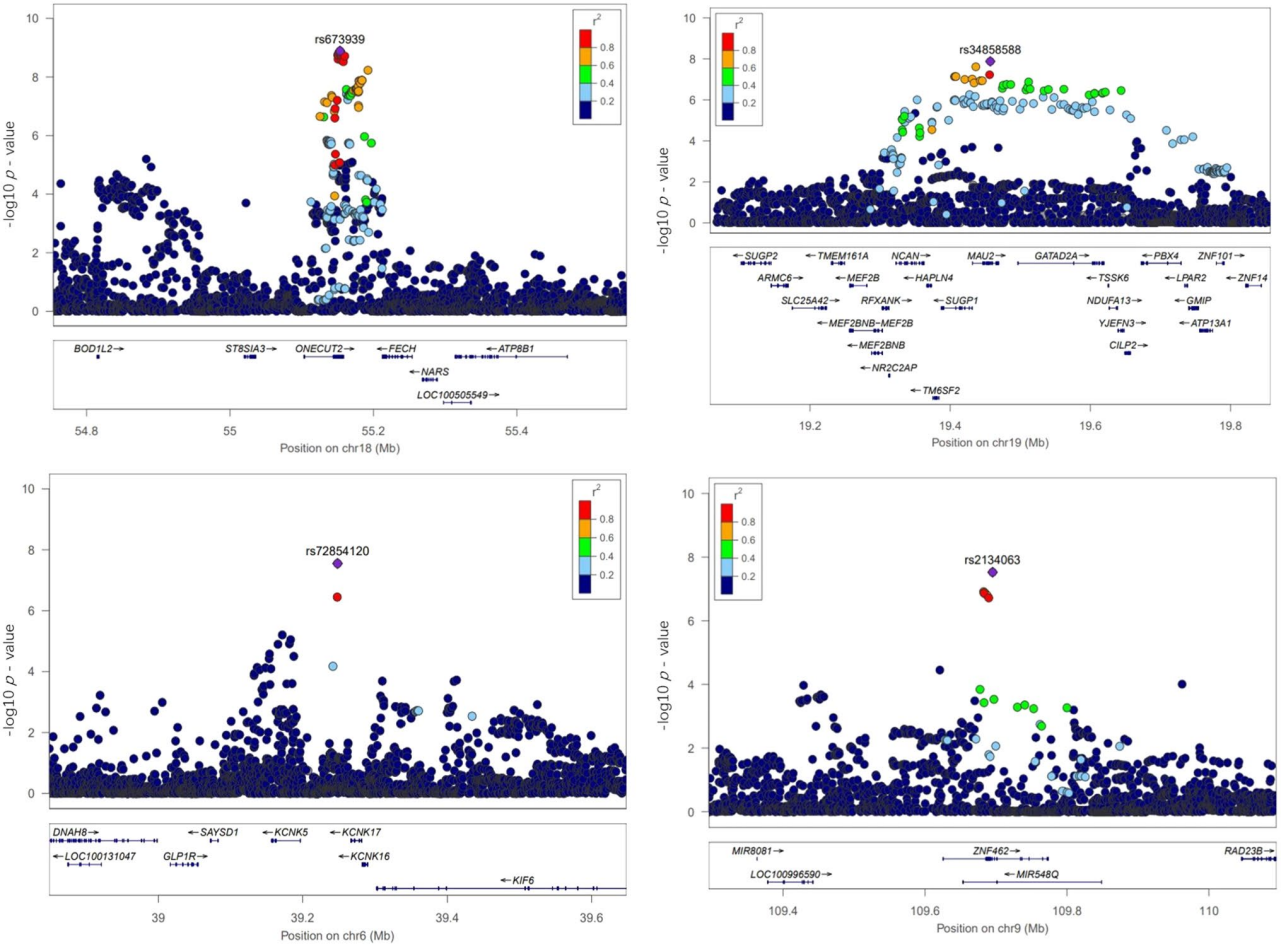
The regional plots of the four new loci. Up left: the *ONECUT2* region; Up right: the *MAU2* region; Bottom left: the Intergenic region (Near *KCNK17*, 6p21.2); Bottom right: the *ZNF462* region

Table 1 summarises the information relating to the 38 associated loci. Among these loci, 25 loci had previously been reported by Gormley et al. (2016), and nine further loci had been separately identified by Meng et al. (2018). Four of the 38 loci were newly suggested. The *One Cut homeobox 2 *(*ONECUT2*) gene locus (18q21.31) was the strongest signal among these four new loci, associated with a *p* value of 1.29 × 10^–9^ for rs673939. Table 2 summarises the details of the four newly suggested loci. Regional plots of these four new loci are included in Fig. 2.

**Table 1:** The 38 loci generated by the GWAS meta-analysis study on self-reported headache and self-reported migraine

Rank	Gene	Lead SNP	Chr	SNP position	*p*-value (migraine)	*p*-value (headache)	Z-value (migraine)	Z-value (headache)	*p-*value (meta)	Reported by Gormley et al. (2016) and the locus rank
1	*LRP1-STAT6-SDR9C7*	rs11172113	12	57,527,283	4.57 × 10^–23^	4.92 × 10^–47^	− 9.87	− 14.41	1.24 × 10^–62^	Yes (1)
2	*FHL5-UFL1*	rs9486715	6	97,059,769	8.69 × 10^–13^	2.58 × 10^–31^	7.16	11.64	6.57 × 10^–39^	Yes (3)
3	*PHACTR1*	rs9349379	6	12,903,957	5.20 × 10^–14^	3.59 × 10^–15^	− 7.51	− 7.87	4.62 × 10^–25^	Yes (6)
4	*TRPM8-HJURP*	rs2362290	2	234,825,369	8.89 × 10^–13^	6.25 × 10^–16^	− 7.11	− 8.08	1.49 × 10^–24^	Yes (5)
5	*PRDM16*	rs10218452	1	3,075,597	1.49 × 10^–18^	4.55 × 10^–9^	8.83	5.86	7.70 × 10^–24^	Yes (2)
6	*MEF2D*	rs2282286	1	156,452,870	5.78 × 10^–10^	2.34 × 10^–11^	6.21	6.68	6.00 × 10^–18^	Yes (7)
7	*Intergenic (Near TSPAN2–NGF)*	rs12134493	1	115,677,946	1.03 × 10^–9^	1.93 × 10^–10^	6.14	6.37	7.48 × 10^–16^	Yes (4)
8	*Intergenic (Near ADAMTSL4–ECM1)*	rs6693567	1	150,510,660	8.11 × 10^–6^	3.77 × 10^–12^	4.47	6.95	7.16 × 10^–15^	Yes (28)
9	*Intergenic (Near GPR149)*	rs34097149	3	154,263,175	6.93 × 10^–7^	1.33 × 10^–10^	− 4.89	− 6.42	2.80 × 10^–14^	Yes (19)
10	*Intergenic (Near FGF6)*	rs10774231	12	4,515,374	4.18 × 10^–8^	4.55 × 10^–9^	− 5.49	− 5.86	3.82 × 10^–14^	Yes (9)
11	*LINC02210-CRHR1-MAPT*	rs117368197	17	43,715,924	7.12 × 10^–1^	2.36 × 10^–14^	− 0.37	7.63	1.08 × 10^–13^	No, reported by Meng et al. (2018)
12	*ASTN2*	rs10759844	9	119,249,326	3.65 × 10^–6^	2.24 × 10^–10^	− 4.62	− 6.34	1.44 × 10^–13^	Yes (13)
13	*SLC24A3*	rs4814864	20	19,469,817	2.36 × 10^–12^	8.88 × 10^–4^	7.03	3.32	2.39 × 10^–13^	Yes (8)
14	*CFDP1*	rs11149826	16	75,435,140	8.82 × 10^–7^	2.85 × 10^–8^	− 4.92	− 5.55	3.20 × 10^–12^	Yes (16)
15	*PLEKHA1 (ARMS2–HTRA1)*	rs78438709	10	124,201,071	9.19 × 10^–3^	8.57 × 10^–12^	− 2.59	− 6.83	1.41 × 10^–11^	Yes (35)
16	*MRVI1*	rs4909945	11	10,673,739	3.93 × 10^–5^	5.13 × 10^–9^	− 4.11	− 5.84	4.58 × 10^–11^	Yes (14)
17	*Intergenic (Near GJA1)*	rs9490318	6	121,860,207	2.27 × 10^–3^	1.72 × 10^–10^	3.06	6.38	6.84 × 10^–11^	Yes (25)
18	*SUGCT*	rs77410344	7	40,410,924	8.45 × 10^–8^	4.84 × 10^–6^	5.39	4.57	7.44 × 10^–11^	Yes (10)
19	*Intergenic (Near JAG1)*	rs6040095	20	10,680,221	7.98 × 10^–8^	4.67 × 10^–6^	5.38	4.58	7.78 × 10^–11^	Yes (20)
20	*RNF213*	rs12943001	17	78,238,645	7.93 × 10^–8^	5.37 × 10^–5^	− 5.36	− 4.04	7.35 × 10^–10^	Yes (17)
21	*ONECUT2*	rs673939	18	55,153,266	4.88 × 10^–5^	1.63 × 10^–7^	− 4.05	− 5.24	1.29 × 10^–9^	No (new locus)
22	*NOL4L*	rs159058	20	31,108,108	3.51 × 10^–5^	3.18 × 10^–7^	− 4.14	− 5.11	1.68 × 10^–9^	No, reported by Meng et al. (2018)
23	*Intergenic (Near ZCCHC2)*	rs4941139	18	60,162,791	7.73 × 10^–1^	2.22 × 10^–10^	0.29	6.35	3.38 × 10^–9^	No, reported by Meng et al. (2018)
24	*Intergenic (Near ZCCHC14)*	rs8052831	16	87,578,039	1.15 × 10^–8^	2.86 × 10^–3^	5.72	2.98	3.80 × 10^–9^	Yes (22)
25	*PLCE1*	rs3891783	10	96,015,793	3.85 × 10^–7^	7.53 × 10^–5^	− 5.07	− 3.96	4.10 × 10^–9^	Yes (11)
26	*CARF*	rs72928613	2	203,839,628	2.58 × 10^–5^	1.23 × 10^–6^	− 4.19	− 4.85	4.80 × 10^–9^	Yes (34)
27	*Intergenic (Near ITPK1)*	rs28540738	14	93,591,673	3.56 × 10^–6^	9.60 × 10^–6^	− 4.63	− 4.43	4.95 × 10^–9^	Yes (27)
28	*CHRM4*	rs2067482	11	46,406,767	9.78 × 10^–4^	3.93 × 10^–8^	− 3.29	− 5.49	4.99 × 10^–9^	No, reported by Meng et al. (2018)
29	*CAMK1D*	rs10752269	10	12,692,902	4.20 × 10^–1^	4.87 × 10^–10^	0.81	6.22	7.11 × 10^–9^	No, reported by Meng et al. (2018)
30	*MAU2*	rs34858588	19	19,457,235	3.53 × 10^–4^	3.40 × 10–7	3.59	5.10	1.33 × 10^–8^	No (new locus)
31	*MYO1H*	rs6606710	12	109,848,903	3.86 × 10^–2^	8.44 × 10^–9^	2.07	5.76	2.60 × 10^–8^	No, reported by Meng et al. (2018)
32	*Intergenic (Near KCNK17)*	rs72854120	6	39,248,533	6.22 × 10^–3^	3.99 × 10^–8^	− 2.68	− 5.49	2.81 × 10^–8^	No (new locus)
33	*ZNF462*	rs2134063	9	109,695,139	1.60 × 10^–3^	1.85 × 10^–7^	3.16	5.21	2.98 × 10^–8^	No (new locus)
34	*Intergenic (Near CDKN2C)*	rs7555006	1	51,480,258	9.56 × 10^–2^	5.87 × 10^–9^	1.67	5.82	3.59 × 10^–8^	No, reported by Meng et al. (2018)
35	*LOC101927995 (Near TGFBR2)*	rs6791480	3	30,480,559	7.70 × 10^–5^	4.60 × 10^–6^	3.96	4.58	3.81 × 10^–8^	Yes (26)
36	*TJP2*	rs7850547	9	71,747,208	5.24 × 10^–4^	7.50 × 10^–7^	− 3.47	− 4.95	4.09 × 10^–8^	No, reported by Meng et al. (2018)
37	*NUFIP2*	rs8614	17	27,588,806	2.36 × 10^–1^	4.25 × 10^–9^	1.19	5.87	4.56 × 10^–8^	No, reported by Meng et al. (2018)
38	*Intergenic (Near REST–SPINK2)*	rs781669	4	57,819,794	4.59x^10−3^	1.09 × 10^–7^	2.83	5.31	4.67 × 10^–8^	Yes (21)

Chr: chromosome

The Z values (ratio of effect size to standard error) stand for the specific SNP effect contribution from each cohort

The locus rank reported in final column is based on the study by Gormley et al. (2016)

**Table 2:** The summary statistics of the four newly suggested loci of headaches

New Locus (Chr)	Lead SNP	Effective allele	23andMe (migraine)		UK Biobank (headache)		Joint meta-analysis
			Beta (SE)	*p value*	Beta (SE)	*p value*	*p value*
*ONECUT2 (18q21.31)*	rs673939	C	− 0.04 (0.01)	4.88 × 10^−5^	− 0.0078 (0.001)	1.63 × 10^–7^	1.29 × 10^–9^
*MAU2 (19p13.11)*	rs34858588	G	0.067 (0.019)	3.53 × 10^−4^	0.013 (0.003)	3.40 × 10^–7^	1.33 × 10^–8^
*Intergenic (Near KCNK17,6p21.2)*	rs72854120	C	− 0.23 (0.08)	6.22 × 10^−3^	− 0.047 (0.009)	3.99 × 10^–8^	2.81 × 10^–8^
*ZNF462 (9q31.2)*	rs2134063	G	0.04 (0.01)	1.60 × 10^−3^	0.0099 (0.002)	1.85 × 10^–7^	2.98 × 10^–8^

The gene-based association study identified 51 genes (cut-off *p* value = 0.05/19,436 = 2.57 × 10^–6^) that were associated with headaches, with the *PR/SET domain 16* (*PRDM16*) gene showing the strongest association (*p* value of 7.10 × 10^–15^). All significantly associated genes are summarised in Supplementary Table 3.

The gene-set analysis found that no specific pathway was significantly associated with headaches after Bonferroni correction (cut-off *p* value = 0.05/10,894 = 4.6 × 10^–6^). The top 10 pathways are included in the Supplementary Table 4.

**Fig. 3:**
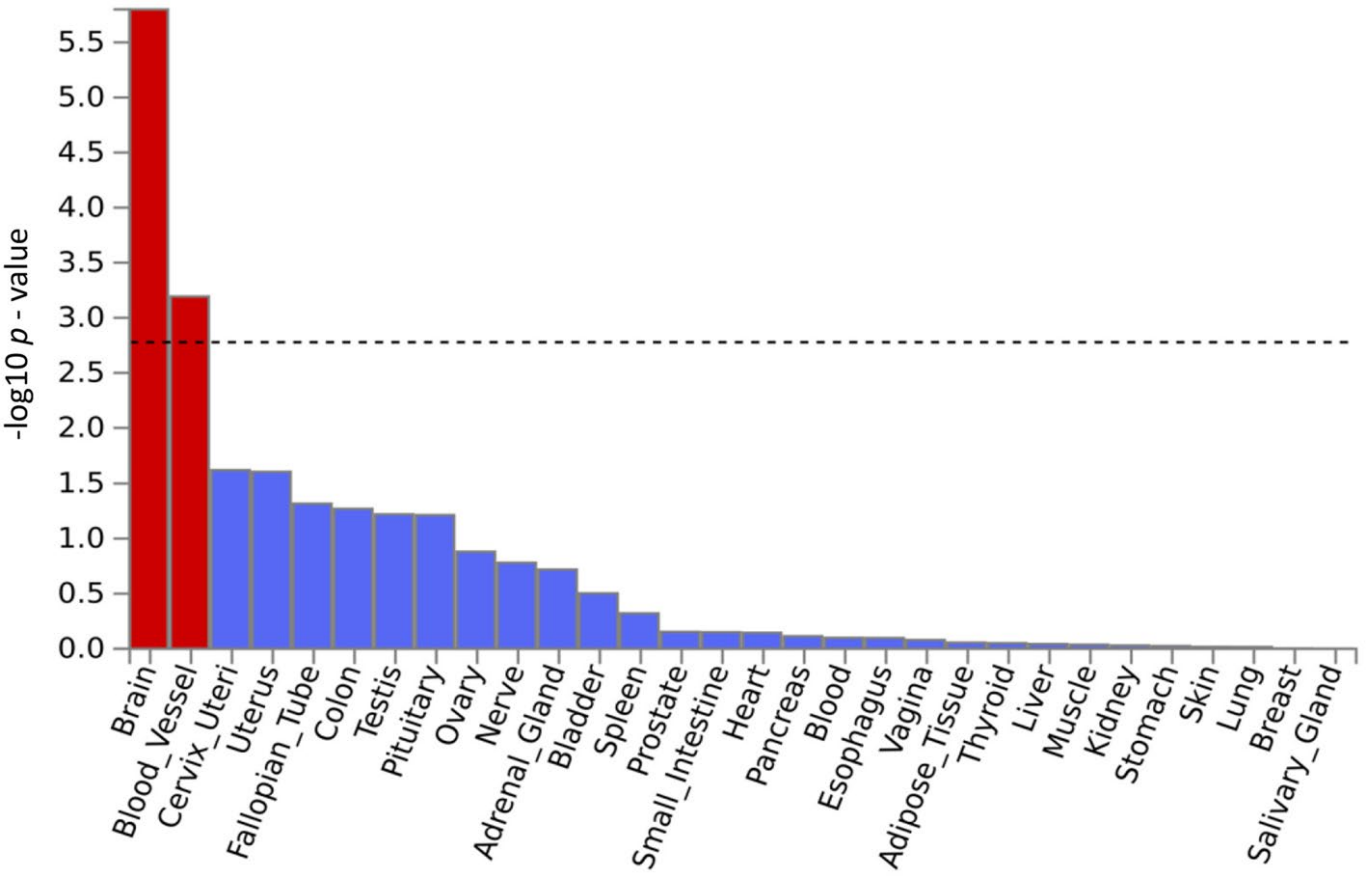
Tissue expression results on 30 specific tissue types by GTEx in the FUMA. The dashed line shows the cut-off *p* value for significance with Bonferroni adjustment for multiple hypothesis testing

**Fig. 4:**
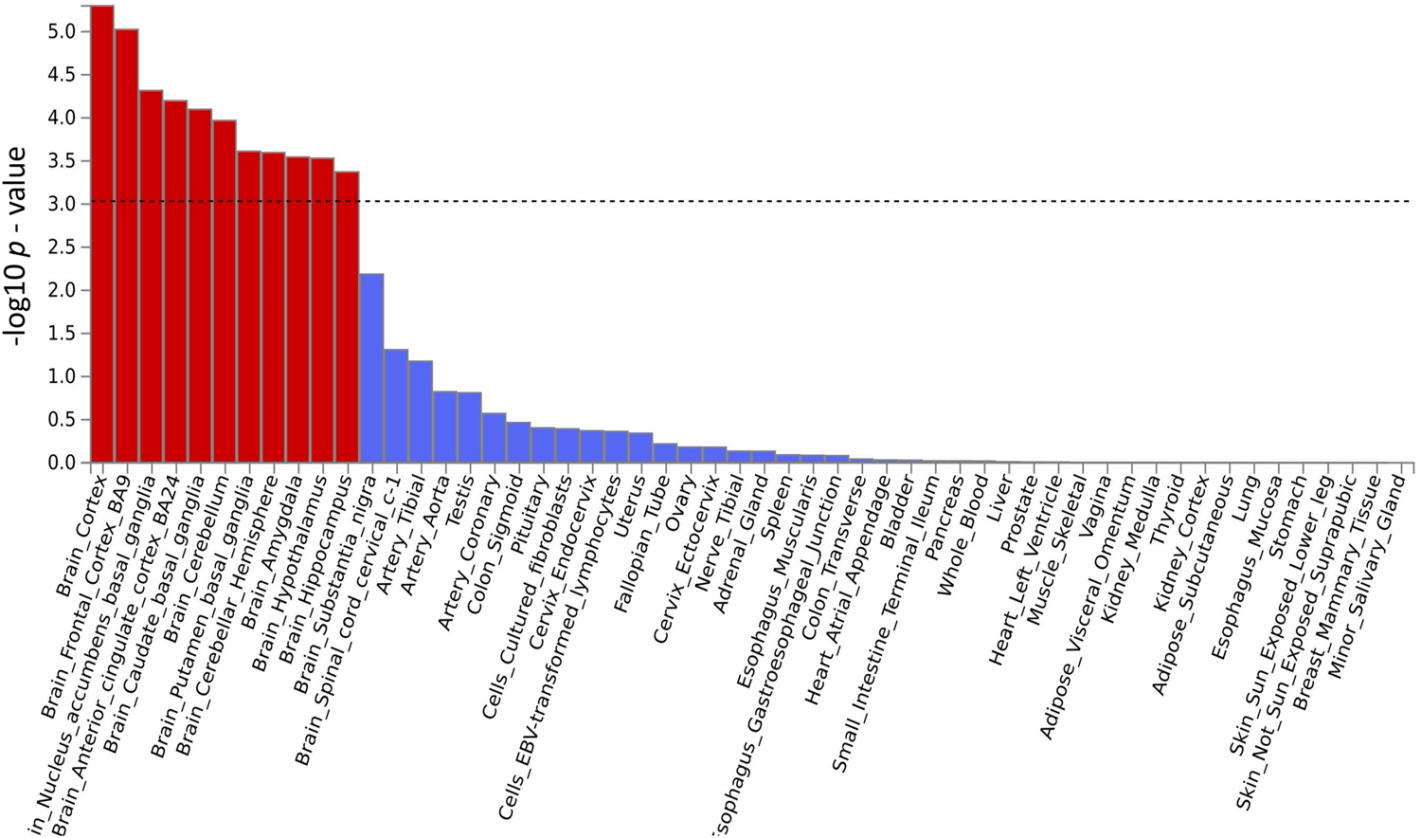
Tissue expression results on 53 specific tissue types by GTEx in the FUMA. The dashed line shows the cut-off *p* value for significance with Bonferroni adjustment for multiple hypothesis testing

Two types of tissue analysis were performed. The tissue expression analysis on 30 general tissues revealed that both brain tissues and vascular tissues are potentially involved in the disease mechanisms (Fig. 3). The tissue expression analysis on 54 specific tissues also found 11 brain tissues with significant association (*p* < 0.05/54 = 9.26 × 10^–4^) (Fig. 4).

We also compared the 28 loci suggested by Meng et al. (2018) with the 38 loci suggested by this study. We noticed that 24 loci reported by Meng et al. (2018) still exist in the current study while four loci dropped out. Fourteen loci showed up in the current study which were not identified by Meng et al. (2018) (Table 3, Supplementary Tables 5 and 6).

**Table 3:** Four loci reported by Meng et al. (2018) while dropped out in the current study

Loci reported by Meng et al	Gene	Chromosome	Lead SNP In Meng et al	SNP position	Still a locus in the current study?
1	*LRP1-STAT6-SDR9C7*	12	rs11172113	57,527,283	Yes
2	*FHL5-UFL1*	6	rs9486715	97,059,769	Yes
3	*TRPM8-HJURP*	2	rs2362290	234,825,369	Yes
4	*PHACTR1*	6	rs9349379	12,903,957	Yes
5	*LINC02210-CRHR1-MAPT*	17	rs77804065	43,810,896	Yes
6	*Intergenic*	1	rs12740679	150,262,270	Yes
7	*Intergenic*	10	rs78438709	124,201,071	Yes
8	*MEF2D*	1	rs1050316	156,434,703	Yes
9	*ASTN2*	9	rs17220352	119,248,059	Yes
10	*Intergenic*	3	rs34097149	154,263,175	Yes
11	*Intergenic*	6	rs9490318	121,860,207	Yes
12	*Intergenic*	1	rs12134493	115,677,946	Yes
13	*Intergenic*	18	rs4941139	60,162,791	Yes
14	*CAMK1D*	10	rs2895526	12,726,061	Yes
15	*PRDM16*	1	rs56304645	3,085,186	Yes
16	*NUFIP2*	17	rs8614	27,588,806	Yes
17	*Intergenic*	12	rs10774231	4,515,374	Yes
18	*MRVI1*	11	rs4909945	10,673,739	Yes
19	*BTN2A2*	6	rs2072806	26,385,093	No
20	*Intergenic*	1	rs7555006	51,480,258	Yes
21	*MYO1H*	12	rs6606710	109,848,903	Yes
22	*IFT81*	12	rs7300001	110,581,731	No
23	*NOL4L*	20	rs1555132	31,046,567	Yes
24	*CFDP1*	16	rs1011121	75,325,933	Yes
25	*PTBP2*	1	rs3748784	97,187,174	No
26	*FXN*	9	rs4596713	71,699,216	Yes
27	*ATG13*	11	rs56349329	46,695,483	Yes
28	*MACF1*	1	rs2036465	39,575,982	No

With the specific permission obtained from the 23andMe, we have included the summary results of the GWAS on self-reported migraine using the 23andMe data in a supplementary file. We also provided a loci comparison table among the four studies (the current study, Gormley et al. 2016; Meng et al. 2018 and 23andMe) (Supplementary Table 7).

## Discussion

We performed a GWAS meta-analysis study on two highly genetically correlated phenotypes based on summary statistics from two large GWAS: self-reported headache and self-reported migraine (genetic correlation value = 0.72, *p* = 1.66 × 10^–68^, standard error = 0.04). This analysis identified 38 loci associated with headaches, of which 34 had been previously identified (Gormley et al. 2016; Meng et al. 2018) and four were newly suggested loci.

GWAS on complex traits have achieved great success in the past decade (Mills and Rahal 2019). Furthermore, GWAS meta-analysis on same phenotypes from multi-centers and multi-cohorts also improve statistical power to identify genetic variants which otherwise cannot be detected by a single cohort study (Evangelou and Ioannidis 2013). However, the number of cohorts in a genetic consortium will reach a bottleneck when most of the existing cohorts are already included. It becomes more difficult to include extra cohorts into the consortium to achieve a higher study power. Meanwhile, it might also be challenging to fund and allocate resources to increase sample size of cohorts in a genetic consortium. This has led to the development and use of statistical methods that leverage other aspects of a study to increase detection power. Software developed for joint meta-analysis of GWAS on existing correlated phenotypes can improve power with minimum additional resource requirement, particularly as it is now a routine requirement for GWAS summary statistics to be shared publicly after publication (Guo and Wu 2019). Specific software created for this purpose includes Software for Correlated Phenotype Analysis (META-SCOPA), Canonical Correlation Analysis (metaCCA), and Multi-Trait Analysis of GWAS (MTAG) (Cichonska et al. 2016; Mägi et al. 2017; Turley et al. 2018).

In this study, we used a recently developed software called metaUSAT, whose properties have been illustrated using simulated data, and the Type 2 Diabetes Genetic Exploration by Next-generation sequencing in multi-Ethnic Samples (T2D-GENES) and the METabolic Syndrome In Men (METSIM) datasets (Fuchsberger et al. 2016; Stancáková et al. 2009). The software does not generate an overall Beta or standard error for each SNP but it calculates a Z value representing the contribution of each SNP from each cohort or each phenotype. As with Beta values, if Z values are both positive or both negative, then the direction of the SNP effect on the traits is the same. In Table 2, we see that the most strongly associated SNPs in the top nine loci all have the same direction of effect. However, the lead SNP in the *Long Intergenic Non-Protein Coding RNA 2210* (*LINC02210*)—*Corticotropin Releasing Hormone Receptor 1* (*CRHR1*)—*Microtubule Associated Protein Tau* (*MAPT*) locus (ranked 10^th^), although significantly associated, showed a different direction of effect in the two datasets. This might indicate that the role of this locus might be different in these two phenotypes. It is possible that a locus could contribute to non-migraine type headaches while contributing minimally to migraine. However, this assumption definitely needs further lab evidence. Comparing the Z-values could be a novel way to differentiate the genetic impact of certain SNPs in genetically correlated yet different phenotypes (Ray and Chatterjee 2020).

Consistent with previous studies, the most significantly associated locus in the *LRP1-STAT6-SDR9C7* region was the strongest locus identified in the meta-analysis (*p* = 1.24 × 10^–62^ for rs11172113) (Gormley et al. 2016; Meng et al. 2018). This locus, ranging from 57,244,168 to 57,629,608 in chromosome 12, contained 122 SNPs associated with genome-wide significance, among the 166 SNPs in the output dataset. The *LRP1* gene has been well established as a migraine gene (Anttila et al. 2010, 2013). One theory about its possible link with migraine is that the LRP1 protein interacts with the glutamate receptors on neurons while the pathophysiology of migraine has been suggested to be related with the glutamate homeostasis (Andreou and Goadsby 2009). The gene-based association study revealed that the *PRDM16* gene was the most significantly associated gene, followed by *CRHR1*, *MAPT*, and *KAT8 regulatory NSL complex subunit 1* (*KANSL1*) (Supplementary Table 3). Through the tissue expression analysis, both brain and vascular tissues were indicated as being involved in the mechanisms of headaches. Gormley et al. (2016) found that vascular factors played a main role in migraine, while in our UK Biobank study, we found that neural tissues were major factors in self-reported headache. We, therefore, deduce that for other types of headaches, such as tension-type headache which produces most headaches in the general population, the role of neural tissue is likely to be greater than that of vascular factors. The cortex has demonstrated the strongest link with headache in the tissue expression analysis. It has been reported that migraine is associated with the changes in cortex functions (Barbanti et al. 2020; Charles and Brennan 2010).

In this study, we suggested four new loci which have not previously been reported to be associated with headaches. The *ONECUT2* gene region was the most strongly associated among these four loci. The Z values from the headache study (*Z* = –5.24) and the migraine study (*Z* = –4.05) were in the same direction. The original *p* values of the top SNP (rs673939) in this region were found to be 9.00 × 10^–8^ by Meng et al. (2018) and 4.88 × 10^–5^ in the 23andMe migraine dataset (Gormley et al. 2016). The *ONECUT2* gene, also termed *OC‑2*, is a newly discovered member of the ONECUT transcription factor family (Jacquemin et al. 1999). *ONECUT2* can widely regulate the protein expression associated with cell proliferation, migration, adhesion, and differentiation, thus being involved in the regulation of the development of an organism (Yu et al. 2020). It has been well reported for its associations with multiple cancers. Although we do not know why it is statistically associated with headaches, the gene is expressed in the brain (https://www.ncbi.nlm.nih.gov/gene/9480). It is not uncommon that SNPs can be associated with multiple phenotypes which seem completely unrelated (Solovieff et al. 2013). The *MAU2 sister chromatid cohesion factor* (*MAU2*) is a protein-coding gene, which plays an important role when cohesions (chromosome-associated multi-subunit protein complex) try to bind to DNA to carry out a large spectrum of chromatin-related functions, including sister chromatid cohesion, DNA repair, transcriptional regulation, and three-dimensional organization of chromatin (Zhu and Wang 2019). Mutations of *MAU2* have been linked with a rare disorder of Cornelia de Lange Syndrome (Parenti et al. 2020). The *Potassium* *two pore domain* *channel subfamily K* *member 17* (*KCNK17*) is the nearest gene to the leading SNP of rs72854120 in the third new locus. Variants of this gene have been reported to be associated with ischaemic stroke, cerebral hemorrhage, and arrhythmia (Friedrich et al. 2014; He et al. 2014). The protein products of *Zinc* *Finger protein 462* (*ZNF462*) have shown important roles in embryonic development in animal models (Cosemans et al. 2018). Variants of this gene have been reported to contribute to craniofacial and neurodevelopmental abnormalities (Weiss et al. 2017). It is worth noting that the *Z* values of each leading SNP in the four new loci were all in the same direction in each of the two cohorts. We also noted that Meng et al. (2018) suggested 28 loci associated with self-reported headache; with 24 of these loci still found in the current study while four dropped out (Table 3), which means we found 14 newly suggested loci when performing the meta-analysis with the 23andMe data in the current study. One reason for dropping the four previously suggested loci might be that the studies are based on the genetically correlated samples but are examining different phenotypes. Of note is that the *p* values of the dropped loci in Meng et al. (2018) could be considered as of marginal GWAS significance, and further work needs to be done to explore their relevance.

Notably, we had particular advantages in this study that would be important to consider if applying these methods to other phenotypes and or samples. One was that the two phenotypes we chose (self-reported headache and migraine) are highly genetically correlated. However, the ability of a study to detect more new variants would be reduced if the two phenotypes are not so highly correlated. Second, our two cohorts were both of mainly European descent and with minimum sample overlap; therefore, we avoided some negative impact (such as increasing type I and type II errors) which could be caused by these factors in the study. There are limitations associated with this approach, being novel in its application. For example, although we successfully addressed phenotypes and datasets which are highly genetically correlated, there are insufficient published studies to allow us to determine the strength of correlation which is required to allow this approach for future studies. This will require our approach to be replicated with other phenotypes and datasets, followed by formal statistical appraisal of the results. Similarly, although it was not directly relevant in our study, consideration will need to be given to applying this approach when there is sample overlapping. It is worth mentioning that the genetic correlation between diagnosed cluster headache phenotype from a mixed UK and Swedish cohort and self-reported headache from the UK Biobank was 0.50 (O’Connor et al. 2021). At the time we conducted this analysis, the four novel loci we suggested were previously unknown. During the preparation stage of this paper, a large GWAS meta-analysis on migraine has been published suggesting 123 migraine-related loci (Hautakangas et al. 2022). Among our four newly suggested loci, three loci have been reported (*ONECUT2*, *MAU2* and *ZNF462)*. Although that study was specifically addressing migraine, rather than the more general headache phenotype that we addressed, this overlap between the new loci suggested in the two studies both helps to confirm the findings of Hautakangas et al. (2022), and the success of our methodological approach. The loci near *KCNK17* on chromosome 6p21.2 was not clearly reported and its nearest loci in the paper was *potassium two pore domain channel subfamily K member 5* (*KCNK5*) which is 60 kb away from *KCNK17*. A recent study on rare variants of migraine showed that significant cis-expression quantitative trait loci (eQTL) in the polycomb response elements (regulatory sites that mediate the silencing of homeotic and other genes) mapped to the *KCNK17* (Techlo et al. 2020).

It is also important to note that the control definitions in the two GWAS datasets were different. The controls used in the UK Biobank self-reported headache phenotype reported no pain within previous month, while the controls used in the 23andMe self-reported migraine phenotype could have had pain in body sites other than the head. This mean that genes identified in the UK Biobank cohort may not be specific to headache, but could be more generally associated with pain. Please also note that self-reported headache or migraine phenotypes are different from clinical ascertained phenotypes. Although our results on self-reported phenotypes will provide reference values to other researchers, there could be potential biases because of this and the results should be interpreted with caution in a clinical setting.

## Conclusion

In summary, our study suggested four new genetic loci which are associated with self-reported headaches and/or migraine, and shed further light on their potential mechanisms. Further research could attempt a meta-analysis study on GWAS of different types of primary headaches (on the condition that they are reasonably genetically correlated) to identify further genetic components.

## Supplementary Information

Below is the link to the electronic supplementary material.

Supplementary file1 (PPTX 89 KB)

Supplementary file2 (DOCX 181 KB)

Supplementary file3 (XLSX 18 KB)

Supplementary file4 (XLSX 141 KB)

Supplementary file5 (XLSX 15 KB)

Supplementary file6 (XLSX 12 KB)

Supplementary file7 (DOCX 16 KB)

Supplementary file8 (DOCX 23 KB)

Supplementary file9 (DOCX 26 KB)

## Data Availability

The GWAS meta-analysis summary statistics can be downloaded from https://app.box.com/s/gm2qkf17hc9w1fc5ymvifgbxz0httvhs. The FUMA results can be viewed from https://fuma.ctglab.nl/browse/334. Data from 23andMe were obtained under a data transfer agreement. Further information about obtaining access to the 23andMe Inc. summary statistics is available from: https://research.23andme.com/collaborate/. Any other data relevant to the study that are not included in the article or its supplementary materials are available from the authors upon reasonable request.
