# Supplementary figures and images for: A Meta-Analysis of the Genome-Wide Association Studies on Two Genetically Correlated Phenotypes Suggests Four New Risk Loci for Headaches
Source: Phenomics. 2022 Nov 18;3(1):64–76. doi: 10.1007/s43657-022-00078-7 (PMC9883337; doi:10.1007/s43657-022-00078-7)

## Slide 1
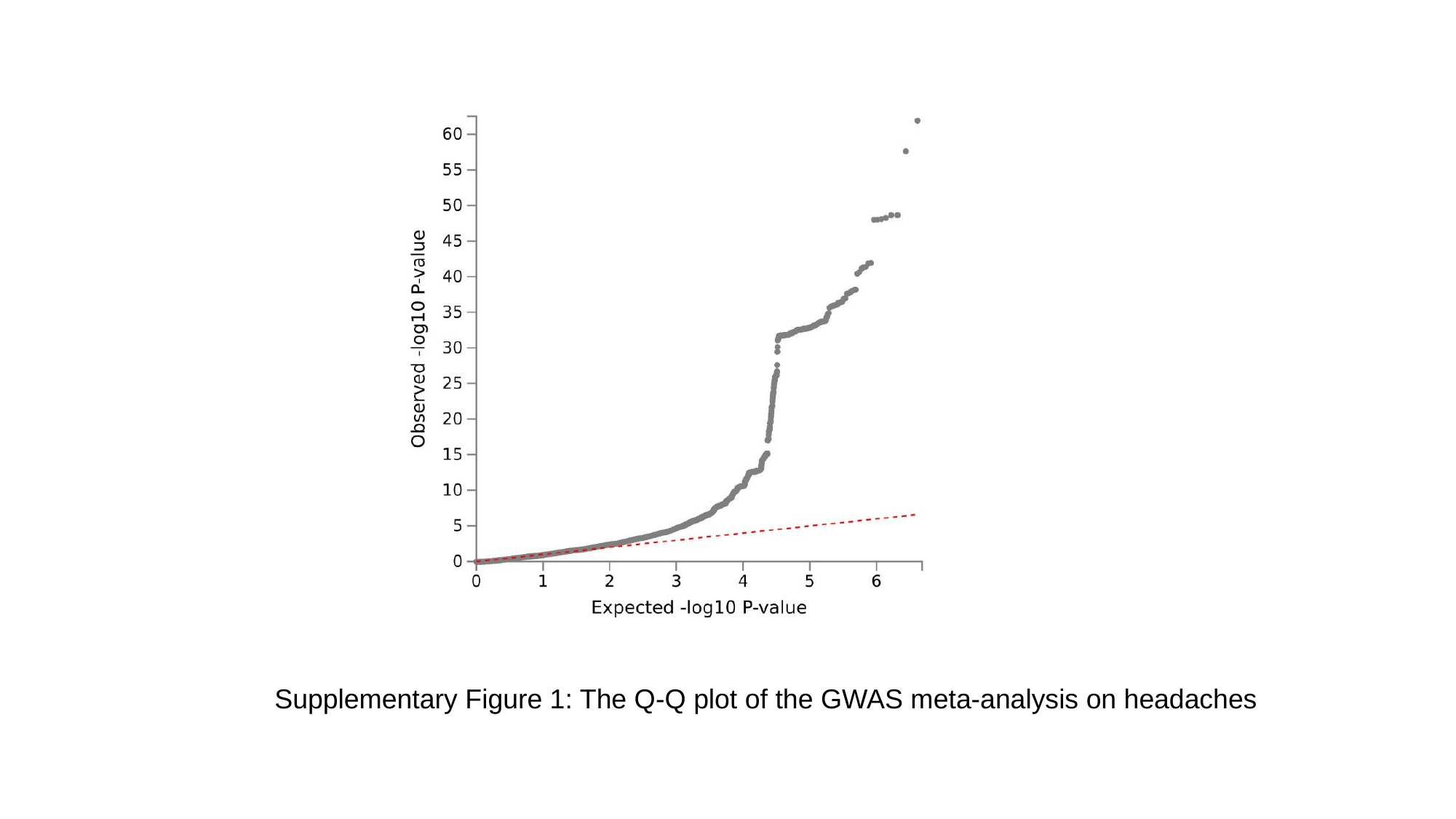

Supplementary Figure 1: The Q-Q plot of the GWAS meta-analysis on headaches

Supplement: Supplementary file 1 — Supplementary file1 (PPTX 89 KB) [file 43657_2022_78_MOESM1_ESM.pptx]
