# Supplementary material for: A Meta-Analysis of the Genome-Wide Association Studies on Two Genetically Correlated Phenotypes Suggests Four New Risk Loci for Headaches
Source: Phenomics. 2022 Nov 18;3(1):64–76. doi: 10.1007/s43657-022-00078-7 (PMC9883337; doi:10.1007/s43657-022-00078-7)
Supplement: Supplementary file 2 — Supplementary file2 (DOCX 181 KB) [file 43657_2022_78_MOESM2_ESM.docx]

Supplementary file: **the summary results of the GWAS on self-reported migraine using the 23andMe data**

The definitions of self-reported migraine (23andMe) were: cases (N=30,465), defined as those who self-reported a migraine history (diagnosed by doctors or self-diagnosing) using the 23andMe online questionnaire; controls (N=143,147), those who self-reported having no migraine. The corresponding GWAS was performed using a linear mixed model adjusting for age, sex, and five population principal components.

The GWAS summary statistics dataset contains 19,023,436 SNPs (containing minor allele frequency and imputation score information for all SNPs). It was uploaded to FUMA for SNP annotation purpose.

1. The Manhattan plot of the GWAS on self-reported migraine using the 23andMe data


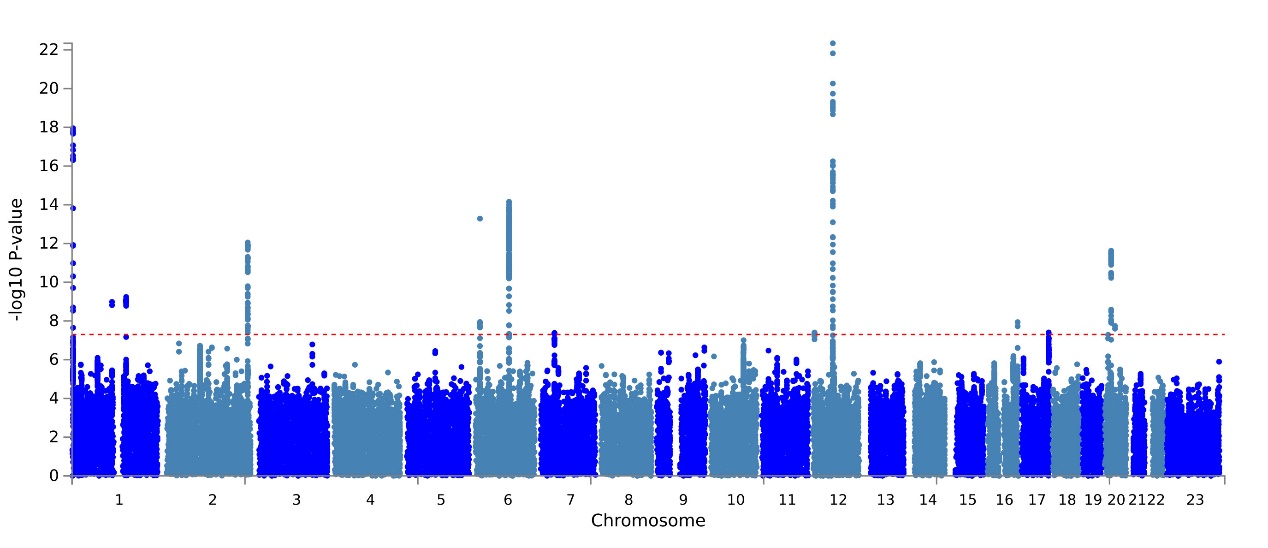


1. All together 13 loci were suggested to be associated with self-reported migraine according to the 23andMe data

| Locus | rsID | Chromosome | Position | p | Gene (or nearest gene) |
| --- | --- | --- | --- | --- | --- |
| 1 | rs61759161 | 1 | 3065568 | 1.12E-18 | *PRDM16* |
| 2 | rs12134493 | 1 | 115677946 | 1.03E-09 | *Intergenic (Near TSPAN2-NGF)* |
| 3 | rs2282286 | 1 | 156452870 | 5.78E-10 | *MEF2D* |
| 4 | rs2362290 | 2 | 234825369 | 8.89E-13 | *TRPM8-HJURP* |
| 5 | rs9349379 | 6 | 12903957 | 5.20E-14 | *PHACTR1* |
| 6 | rs2971603 | 6 | 97035418 | 7.02E-15 | *FHL5-UFL1* |
| 7 | rs146430923 | 7 | 40373767 | 4.14E-08 | *SUGCT* |
| 8 | rs7300066 | 12 | 4529169 | 3.94E-08 | *Intergenic (Near FGF6)* |
| 9 | rs11172113 | 12 | 57527283 | 4.57E-23 | *LRP1-STAT6-SDR9C7* |
| 10 | rs8052831 | 16 | 87578039 | 1.15E-08 | *Intergenic (Near ZCCHC14)* |
| 11 | rs17857135 | 17 | 78262161 | 3.93E-08 | *RNF213* |
| 12 | rs4814864 | 20 | 19469817 | 2.36E-12 | *SLC24A3* |
| 13 | rs76627106 | 20 | 30610164 | 1.82E-08 | *CCM2L* |

1. A list of all 457 SNPs with a *p* value less than 5x 10^-8^ in the GWAS output

| rsID | Chromosome | Position | *p* |
| --- | --- | --- | --- |
| rs11172113 | 12 | 57527283 | 4.57E-23 |
| rs4759276 | 12 | 57526646 | 1.51E-22 |
| rs4759275 | 12 | 57525756 | 5.48E-21 |
| rs11172111 | 12 | 57515804 | 1.83E-20 |
| rs10467124 | 12 | 57519694 | 4.82E-20 |
| rs12298170 | 12 | 57515363 | 6.27E-20 |
| rs12322902 | 12 | 57513866 | 8.21E-20 |
| rs10876963 | 12 | 57514554 | 1.02E-19 |
| rs11172110 | 12 | 57515769 | 1.33E-19 |
| rs12309413 | 12 | 57513879 | 2.13E-19 |
| rs61759161 | 1 | 3065568 | 1.12E-18 |
| rs10218452 | 1 | 3075597 | 1.49E-18 |
| rs10909886 | 1 | 3066761 | 1.90E-18 |
| rs10797381 | 1 | 3072482 | 2.14E-18 |
| rs2075968 | 1 | 3081241 | 8.53E-18 |
| rs7518255 | 1 | 3086464 | 1.48E-17 |
| rs56304645 | 1 | 3085186 | 2.91E-17 |
| rs2376495 | 1 | 3095126 | 4.00E-17 |
| rs1393064 | 1 | 3099138 | 4.47E-17 |
| rs61759167 | 1 | 3091587 | 4.59E-17 |
| rs11587518 | 1 | 3096925 | 4.85E-17 |
| rs28594467 | 1 | 3097051 | 4.86E-17 |
| rs4257011 | 12 | 57528197 | 5.68E-17 |
| rs10747776 | 12 | 57528679 | 8.76E-17 |
| rs11172114 | 12 | 57529443 | 9.92E-17 |
| rs1385526 | 12 | 57532749 | 2.04E-16 |
| rs10876965 | 12 | 57531450 | 2.57E-16 |
| rs1799737 | 12 | 57534912 | 3.08E-16 |
| rs10876964 | 12 | 57530814 | 3.08E-16 |
| rs1466535 | 12 | 57534470 | 3.89E-16 |
| rs4759045 | 12 | 57534641 | 5.07E-16 |
| rs4759277 | 12 | 57533690 | 7.27E-16 |
| rs4367982 | 12 | 57531632 | 7.46E-16 |
| rs11172106 | 12 | 57512875 | 1.19E-15 |
| rs12312693 | 12 | 57511734 | 1.66E-15 |
| rs73120411 | 12 | 57519826 | 1.97E-15 |
| rs3001426 | 12 | 57509055 | 6.16E-15 |
| rs2971603 | 6 | 97035418 | 7.02E-15 |
| rs324014 | 12 | 57510309 | 8.88E-15 |
| rs2983896 | 6 | 97029871 | 1.42E-14 |
| rs11153071 | 6 | 97039741 | 1.43E-14 |
| rs2983897 | 6 | 97033780 | 1.66E-14 |
| rs2971606 | 6 | 97043025 | 1.98E-14 |
| rs3860231 | 6 | 97036563 | 2.33E-14 |
| rs3860229 | 6 | 97036559 | 2.34E-14 |
| rs2971608 | 6 | 97039757 | 2.36E-14 |
| rs3860230 | 6 | 97036560 | 2.36E-14 |
| rs3798293 | 6 | 97033370 | 3.31E-14 |
| rs12214469 | 6 | 97019471 | 4.34E-14 |
| rs11153059 | 6 | 97014537 | 4.36E-14 |
| rs11153064 | 6 | 97021959 | 4.43E-14 |
| rs3798289 | 6 | 97023632 | 4.44E-14 |
| rs11153058 | 6 | 97012746 | 4.46E-14 |
| rs12527021 | 6 | 97025496 | 4.47E-14 |
| rs12208124 | 6 | 97027942 | 4.52E-14 |
| rs13202675 | 6 | 97028427 | 4.65E-14 |
| rs10872018 | 6 | 97026429 | 4.85E-14 |
| rs2273622 | 6 | 97058567 | 5.13E-14 |
| rs2145535 | 6 | 97034715 | 5.14E-14 |
| rs9349379 | 6 | 12903957 | 5.20E-14 |
| rs11759769 | 6 | 97065212 | 5.21E-14 |
| rs9386648 | 6 | 97035656 | 5.22E-14 |
| rs11751075 | 6 | 97065751 | 5.24E-14 |
| rs12204068 | 6 | 97036201 | 5.25E-14 |
| rs11756315 | 6 | 97008678 | 5.31E-14 |
| rs6923212 | 6 | 97061940 | 5.54E-14 |
| rs34051754 | 6 | 97007754 | 5.99E-14 |
| rs3798294 | 6 | 97049587 | 6.33E-14 |
| rs13206547 | 6 | 97006942 | 6.45E-14 |
| rs9373978 | 6 | 97056255 | 6.76E-14 |
| rs12197411 | 6 | 97039621 | 6.78E-14 |
| rs4486027 | 6 | 96911886 | 6.82E-14 |
| rs12525695 | 6 | 97039818 | 7.13E-14 |
| rs3906199 | 6 | 97049899 | 7.20E-14 |
| rs11153067 | 6 | 97039421 | 7.20E-14 |
| rs12204342 | 6 | 97054314 | 7.32E-14 |
| rs11153023 | 6 | 96968525 | 7.86E-14 |
| rs12208011 | 6 | 96991105 | 7.91E-14 |
| rs10457159 | 6 | 96993139 | 7.96E-14 |
| rs324012 | 12 | 57502981 | 7.97E-14 |
| rs3734238 | 6 | 96996228 | 8.16E-14 |
| rs3734239 | 6 | 96996244 | 8.18E-14 |
| rs6921291 | 6 | 97066242 | 8.52E-14 |
| rs12530462 | 6 | 97053514 | 8.63E-14 |
| rs3798296 | 6 | 97052286 | 8.98E-14 |
| rs3798295 | 6 | 97052164 | 9.01E-14 |
| rs9486719 | 6 | 97060124 | 9.15E-14 |
| rs13210597 | 6 | 96969694 | 9.34E-14 |
| rs6916232 | 6 | 96985959 | 9.70E-14 |
| rs12207570 | 6 | 96987884 | 9.78E-14 |
| rs2971609 | 6 | 97039665 | 1.10E-13 |
| rs72932805 | 6 | 96902933 | 1.34E-13 |
| rs3849198 | 6 | 97036539 | 1.55E-13 |
| rs4265039 | 6 | 96878929 | 2.02E-13 |
| rs4346856 | 6 | 96880270 | 2.03E-13 |
| rs6568392 | 6 | 96861476 | 2.20E-13 |
| rs12212614 | 6 | 97036466 | 2.33E-13 |
| rs12201449 | 6 | 96959382 | 2.59E-13 |
| rs11153018 | 6 | 96956137 | 2.60E-13 |
| rs12193732 | 6 | 96942609 | 2.79E-13 |
| rs112107820 | 6 | 96943946 | 2.83E-13 |
| rs10447413 | 6 | 96943434 | 2.86E-13 |
| rs146759159 | 6 | 96944004 | 2.88E-13 |
| rs9400016 | 6 | 96940883 | 2.90E-13 |
| rs4262195 | 6 | 96929475 | 3.39E-13 |
| rs12190520 | 6 | 96928620 | 3.41E-13 |
| rs13208321 | 6 | 96860354 | 3.81E-13 |
| rs2499804 | 6 | 96861216 | 4.03E-13 |
| rs9398072 | 6 | 96911304 | 4.03E-13 |
| rs13201795 | 6 | 96910471 | 4.14E-13 |
| rs2499817 | 6 | 96878321 | 4.18E-13 |
| rs2499816 | 6 | 96878206 | 4.18E-13 |
| rs35035182 | 6 | 96885300 | 4.33E-13 |
| rs12199452 | 6 | 96880703 | 4.36E-13 |
| rs11152953 | 6 | 96894305 | 4.47E-13 |
| rs11152952 | 6 | 96894303 | 4.47E-13 |
| rs2472880 | 6 | 96860130 | 4.49E-13 |
| rs11152960 | 6 | 96900398 | 4.52E-13 |
| rs11152959 | 6 | 96900272 | 4.52E-13 |
| rs4839837 | 6 | 96908706 | 4.52E-13 |
| rs28623445 | 6 | 96899116 | 4.53E-13 |
| rs34826652 | 6 | 96899085 | 4.54E-13 |
| rs12190508 | 6 | 96898269 | 4.55E-13 |
| rs12190507 | 6 | 96898265 | 4.56E-13 |
| rs11152970 | 6 | 96909077 | 4.58E-13 |
| rs13202091 | 6 | 96887771 | 4.58E-13 |
| rs11152969 | 6 | 96908712 | 4.60E-13 |
| rs11152968 | 6 | 96908653 | 4.61E-13 |
| rs12195315 | 6 | 96905711 | 4.62E-13 |
| rs4077932 | 6 | 96905144 | 4.64E-13 |
| rs324013 | 12 | 57510661 | 4.64E-13 |
| rs12213426 | 6 | 96896035 | 4.65E-13 |
| rs4078038 | 6 | 96904366 | 4.66E-13 |
| rs12529248 | 6 | 96895377 | 4.69E-13 |
| rs2472881 | 6 | 96862562 | 4.69E-13 |
| rs12210146 | 6 | 96903962 | 4.70E-13 |
| rs12213375 | 6 | 96902134 | 4.71E-13 |
| rs12191653 | 6 | 96903898 | 4.71E-13 |
| rs12208449 | 6 | 96903253 | 4.74E-13 |
| rs75303492 | 6 | 96902999 | 4.80E-13 |
| rs2472882 | 6 | 96863474 | 4.81E-13 |
| rs147506219 | 6 | 96889290 | 4.87E-13 |
| rs140961595 | 6 | 96888323 | 4.88E-13 |
| rs2122692 | 12 | 57510511 | 4.88E-13 |
| rs2472888 | 6 | 96865501 | 4.88E-13 |
| rs2472897 | 6 | 96870569 | 4.89E-13 |
| rs2092096 | 6 | 96870919 | 4.89E-13 |
| rs2092095 | 6 | 96870864 | 4.89E-13 |
| rs13213297 | 6 | 96887569 | 4.89E-13 |
| rs2499809 | 6 | 96870110 | 4.89E-13 |
| rs2142652 | 6 | 96868504 | 4.90E-13 |
| rs2179126 | 6 | 96871350 | 4.90E-13 |
| rs2472895 | 6 | 96870387 | 4.90E-13 |
| rs2142653 | 6 | 96871520 | 4.90E-13 |
| rs2472884 | 6 | 96863965 | 4.91E-13 |
| rs2499813 | 6 | 96875338 | 4.92E-13 |
| rs2472890 | 6 | 96867646 | 4.93E-13 |
| rs11757102 | 6 | 96885073 | 4.93E-13 |
| rs11757063 | 6 | 96884886 | 4.93E-13 |
| rs2499807 | 6 | 96864053 | 4.93E-13 |
| rs11752584 | 6 | 96884952 | 4.93E-13 |
| rs2472908 | 6 | 96875998 | 4.94E-13 |
| rs72931061 | 6 | 96880285 | 4.94E-13 |
| rs67996804 | 6 | 96884387 | 4.94E-13 |
| rs35487471 | 6 | 96879715 | 4.94E-13 |
| rs34236778 | 6 | 96884556 | 4.94E-13 |
| rs4486019 | 6 | 96883814 | 4.95E-13 |
| rs2263192 | 6 | 96873037 | 4.96E-13 |
| rs12203657 | 6 | 96883285 | 4.96E-13 |
| rs13207333 | 6 | 96882254 | 4.97E-13 |
| rs34209750 | 6 | 96882215 | 4.98E-13 |
| rs2472889 | 6 | 96866803 | 5.02E-13 |
| rs2205755 | 6 | 96864704 | 5.07E-13 |
| rs2472911 | 6 | 96877055 | 5.09E-13 |
| rs2472886 | 6 | 96864876 | 5.14E-13 |
| rs11152951 | 6 | 96894135 | 5.16E-13 |
| rs2472904 | 6 | 96874962 | 5.18E-13 |
| rs2472906 | 6 | 96875318 | 5.18E-13 |
| rs2472887 | 6 | 96865437 | 5.24E-13 |
| rs1303852 | 6 | 96864523 | 5.29E-13 |
| rs148083617 | 6 | 96888353 | 5.30E-13 |
| rs12208682 | 6 | 96921307 | 5.56E-13 |
| rs1157316 | 6 | 96869710 | 5.62E-13 |
| rs12194971 | 6 | 96911073 | 5.72E-13 |
| rs6568443 | 6 | 96944473 | 5.95E-13 |
| rs142957763 | 6 | 96890032 | 6.06E-13 |
| rs12205728 | 6 | 96891838 | 6.10E-13 |
| rs13208422 | 6 | 96892119 | 6.10E-13 |
| rs12205655 | 6 | 96891733 | 6.10E-13 |
| rs10457174 | 6 | 97067047 | 6.43E-13 |
| rs12190397 | 6 | 97036465 | 6.54E-13 |
| rs4839833 | 6 | 96882069 | 7.25E-13 |
| rs2499814 | 6 | 96877245 | 7.55E-13 |
| rs2499801 | 6 | 96854594 | 8.22E-13 |
| rs35410524 | 6 | 96885405 | 8.29E-13 |
| rs9486715 | 6 | 97059769 | 8.69E-13 |
| rs2362290 | 2 | 234825369 | 8.89E-13 |
| rs2472883 | 6 | 96863817 | 9.13E-13 |
| rs2273621 | 6 | 97058553 | 9.40E-13 |
| rs760619 | 6 | 96857431 | 1.02E-12 |
| rs7775721 | 6 | 97056979 | 1.03E-12 |
| rs7968719 | 12 | 57540751 | 1.13E-12 |
| rs11153082 | 6 | 97059666 | 1.14E-12 |
| rs6920913 | 6 | 96874297 | 1.14E-12 |
| rs10166942 | 2 | 234825093 | 1.19E-12 |
| rs1965629 | 2 | 234824731 | 1.20E-12 |
| rs1985366 | 2 | 234824559 | 1.20E-12 |
| rs12745073 | 1 | 3094672 | 1.20E-12 |
| rs12208556 | 6 | 96903486 | 1.24E-12 |
| rs10170399 | 2 | 234826648 | 1.25E-12 |
| rs6738979 | 2 | 234823058 | 1.27E-12 |
| rs926276 | 6 | 96868735 | 1.30E-12 |
| rs1003540 | 2 | 234825884 | 1.30E-12 |
| rs1393065 | 1 | 3099105 | 1.31E-12 |
| rs11563063 | 2 | 234821530 | 1.34E-12 |
| rs11153070 | 6 | 97039720 | 1.41E-12 |
| rs9486725 | 6 | 97061159 | 1.45E-12 |
| rs6724624 | 2 | 234820578 | 1.53E-12 |
| rs7741683 | 6 | 97054493 | 1.55E-12 |
| rs10187654 | 2 | 234814059 | 1.62E-12 |
| rs9373907 | 6 | 96981189 | 1.69E-12 |
| rs4663983 | 2 | 234815005 | 1.87E-12 |
| rs12208884 | 6 | 96885572 | 2.05E-12 |
| rs11892538 | 2 | 234821445 | 2.09E-12 |
| rs211158 | 6 | 97022938 | 2.11E-12 |
| rs211159 | 6 | 97023026 | 2.22E-12 |
| rs4814864 | 20 | 19469817 | 2.36E-12 |
| rs4814860 | 20 | 19469395 | 2.64E-12 |
| rs4759044 | 12 | 57530670 | 2.78E-12 |
| rs4814863 | 20 | 19469685 | 2.92E-12 |
| rs9386670 | 6 | 97060688 | 3.29E-12 |
| rs6035354 | 20 | 19464926 | 3.31E-12 |
| rs4814858 | 20 | 19459328 | 3.41E-12 |
| rs9486205 | 6 | 96877006 | 3.48E-12 |
| rs4814861 | 20 | 19469534 | 3.61E-12 |
| rs2255552 | 6 | 96999643 | 3.91E-12 |
| rs211186 | 6 | 97048982 | 4.21E-12 |
| rs6046134 | 20 | 19462636 | 4.40E-12 |
| rs6035353 | 20 | 19462207 | 4.52E-12 |
| rs9486467 | 6 | 96971803 | 4.75E-12 |
| rs7604471 | 2 | 234834176 | 4.93E-12 |
| rs4598081 | 6 | 96975227 | 5.08E-12 |
| rs4233636 | 2 | 234834402 | 5.28E-12 |
| rs4663986 | 2 | 234834455 | 5.35E-12 |
| rs6081612 | 20 | 19460951 | 5.43E-12 |
| rs3849199 | 6 | 97049191 | 5.51E-12 |
| rs2475023 | 6 | 96996833 | 5.64E-12 |
| rs737636 | 2 | 234832824 | 5.69E-12 |
| rs6046139 | 20 | 19474337 | 5.77E-12 |
| rs211187 | 6 | 97036142 | 5.80E-12 |
| rs6431648 | 2 | 234833457 | 5.80E-12 |
| rs1984571 | 20 | 19475621 | 5.82E-12 |
| rs2983902 | 6 | 97047322 | 6.29E-12 |
| rs2971605 | 6 | 97047146 | 6.66E-12 |
| rs1475748 | 6 | 97034143 | 6.81E-12 |
| rs6046140 | 20 | 19476530 | 6.90E-12 |
| rs2499775 | 6 | 97007340 | 6.93E-12 |
| rs2499774 | 6 | 97006581 | 7.02E-12 |
| rs1127175 | 6 | 96999725 | 7.03E-12 |
| rs6709005 | 2 | 234828723 | 7.04E-12 |
| rs2971602 | 6 | 97022468 | 7.28E-12 |
| rs6046137 | 20 | 19466832 | 7.48E-12 |
| rs6136756 | 20 | 19476671 | 7.53E-12 |
| rs1855533 | 6 | 97050265 | 7.67E-12 |
| rs6035357 | 20 | 19478696 | 8.61E-12 |
| rs2362292 | 2 | 234836770 | 8.85E-12 |
| rs6899647 | 6 | 97065661 | 9.83E-12 |
| rs2025044 | 6 | 97067028 | 1.04E-11 |
| rs12368672 | 12 | 57512470 | 1.05E-11 |
| rs4131519 | 6 | 96964939 | 1.06E-11 |
| rs3827986 | 20 | 19475014 | 1.08E-11 |
| rs6046144 | 20 | 19477390 | 1.15E-11 |
| rs2971607 | 6 | 97039857 | 1.16E-11 |
| rs6905568 | 6 | 97066382 | 1.23E-11 |
| rs3798297 | 6 | 97062835 | 1.26E-11 |
| rs11153085 | 6 | 97066355 | 1.26E-11 |
| rs6046147 | 20 | 19479796 | 1.27E-11 |
| rs9486373 | 6 | 96942450 | 1.37E-11 |
| rs6905737 | 6 | 97066659 | 1.39E-11 |
| rs4240553 | 6 | 96965324 | 1.39E-11 |
| rs6902835 | 6 | 97063005 | 1.43E-11 |
| rs9384628 | 6 | 96960232 | 1.44E-11 |
| rs2475025 | 6 | 96988696 | 1.46E-11 |
| rs9386576 | 6 | 96954717 | 1.46E-11 |
| rs9400034 | 6 | 96954566 | 1.46E-11 |
| rs4839687 | 6 | 96951732 | 1.48E-11 |
| rs9373985 | 6 | 97063522 | 1.49E-11 |
| rs10189040 | 2 | 234843191 | 1.51E-11 |
| rs758275 | 2 | 234838020 | 1.52E-11 |
| rs9398148 | 6 | 97063555 | 1.62E-11 |
| rs6711120 | 2 | 234845134 | 1.65E-11 |
| rs4839840 | 6 | 96941149 | 1.69E-11 |
| rs9480700 | 6 | 96965056 | 1.69E-11 |
| rs2499777 | 6 | 97013814 | 1.71E-11 |
| rs9486444 | 6 | 96963619 | 1.74E-11 |
| rs12207471 | 6 | 96963782 | 1.74E-11 |
| rs4839842 | 6 | 96955387 | 1.81E-11 |
| rs4518508 | 6 | 96890450 | 1.87E-11 |
| rs9372106 | 6 | 96875058 | 1.88E-11 |
| rs2108810 | 2 | 234845708 | 1.90E-11 |
| rs4343922 | 6 | 96876903 | 2.07E-11 |
| rs9386473 | 6 | 96873341 | 2.07E-11 |
| rs7132082 | 12 | 57366210 | 2.09E-11 |
| rs4438964 | 6 | 96917563 | 2.15E-11 |
| rs758276 | 2 | 234837808 | 2.29E-11 |
| rs1009596 | 6 | 96843688 | 2.45E-11 |
| rs9646720 | 2 | 234847573 | 2.48E-11 |
| rs9384585 | 6 | 96875664 | 2.57E-11 |
| rs9373829 | 6 | 96897201 | 2.58E-11 |
| rs9384602 | 6 | 96919561 | 2.59E-11 |
| rs9486275 | 6 | 96901187 | 2.75E-11 |
| rs7452350 | 6 | 96883326 | 2.76E-11 |
| rs11152924 | 6 | 96877383 | 2.76E-11 |
| rs6928442 | 6 | 96870118 | 2.78E-11 |
| rs926277 | 6 | 96868837 | 2.78E-11 |
| rs9486181 | 6 | 96868132 | 2.79E-11 |
| rs7451950 | 6 | 96879974 | 2.82E-11 |
| rs4425602 | 6 | 96893906 | 2.83E-11 |
| rs9373833 | 6 | 96904998 | 2.84E-11 |
| rs9373887 | 6 | 96957004 | 2.86E-11 |
| rs2302153 | 2 | 234847823 | 2.94E-11 |
| rs10490012 | 2 | 234843494 | 3.04E-11 |
| rs2223238 | 6 | 96871855 | 3.23E-11 |
| rs6081613 | 20 | 19465907 | 3.26E-11 |
| rs6035355 | 20 | 19465089 | 3.42E-11 |
| rs4839828 | 6 | 96863326 | 3.43E-11 |
| rs6568428 | 6 | 96903604 | 3.46E-11 |
| rs9373818 | 6 | 96885823 | 3.51E-11 |
| rs4839834 | 6 | 96882109 | 3.52E-11 |
| rs9386500 | 6 | 96889845 | 3.52E-11 |
| rs3860243 | 6 | 96905303 | 3.53E-11 |
| rs9320127 | 6 | 96865358 | 3.86E-11 |
| rs3790228 | 20 | 19469150 | 4.30E-11 |
| rs3058639 | 20 | 19466843 | 4.32E-11 |
| rs2499787 | 6 | 96841762 | 4.56E-11 |
| rs2205754 | 6 | 96843134 | 4.64E-11 |
| rs1009597 | 6 | 96843540 | 4.70E-11 |
| rs2499792 | 6 | 96846264 | 4.78E-11 |
| rs2472875 | 6 | 96844691 | 4.79E-11 |
| rs2472876 | 6 | 96845723 | 4.83E-11 |
| rs2499793 | 6 | 96846402 | 4.84E-11 |
| rs2499790 | 6 | 96845078 | 4.91E-11 |
| rs2499797 | 6 | 96848669 | 5.13E-11 |
| rs976357 | 6 | 96849679 | 5.13E-11 |
| rs2499796 | 6 | 96848364 | 5.17E-11 |
| rs976356 | 6 | 96849437 | 5.19E-11 |
| rs2049927 | 6 | 96848958 | 5.22E-11 |
| rs2499799 | 6 | 96851676 | 5.81E-11 |
| rs4759042 | 12 | 57377347 | 5.92E-11 |
| rs3790227 | 20 | 19469002 | 5.96E-11 |
| rs2092094 | 6 | 96844123 | 6.06E-11 |
| rs2103539 | 6 | 96844342 | 6.13E-11 |
| rs2499789 | 6 | 96844510 | 6.34E-11 |
| rs1843314 | 12 | 57362422 | 1.48E-10 |
| rs2362293 | 2 | 234836854 | 1.61E-10 |
| rs13418652 | 2 | 234845580 | 1.78E-10 |
| rs9646719 | 2 | 234845571 | 2.00E-10 |
| rs9386528 | 6 | 96928514 | 2.15E-10 |
| rs3122929 | 12 | 57509102 | 3.10E-10 |
| rs3001427 | 12 | 57508649 | 3.25E-10 |
| rs9646718 | 2 | 234830440 | 3.95E-10 |
| rs12988953 | 2 | 234826661 | 4.45E-10 |
| rs7770889 | 6 | 96858453 | 5.32E-10 |
| rs1004478 | 2 | 234825895 | 5.65E-10 |
| rs2282286 | 1 | 156452870 | 5.78E-10 |
| rs3001425 | 12 | 57509569 | 7.45E-10 |
| rs3818463 | 1 | 156453441 | 7.75E-10 |
| rs2274320 | 1 | 156453247 | 7.84E-10 |
| rs11264486 | 1 | 156455173 | 8.10E-10 |
| rs1925950 | 1 | 156450740 | 8.18E-10 |
| rs6700679 | 1 | 156442956 | 8.20E-10 |
| rs3790454 | 1 | 156451349 | 8.48E-10 |
| rs6658120 | 1 | 156448426 | 8.62E-10 |
| rs4450010 | 1 | 156448209 | 8.94E-10 |
| rs12131289 | 1 | 156446450 | 9.00E-10 |
| rs2274316 | 1 | 156446242 | 9.02E-10 |
| rs10908505 | 1 | 156468243 | 9.33E-10 |
| rs3790455 | 1 | 156456301 | 9.36E-10 |
| rs1342442 | 1 | 156466699 | 9.93E-10 |
| rs12134493 | 1 | 115677946 | 1.03E-09 |
| rs1050316 | 1 | 156434703 | 1.07E-09 |
| rs12136856 | 1 | 156473114 | 1.09E-09 |
| rs3790459 | 1 | 156461707 | 1.10E-09 |
| rs10908504 | 1 | 156464429 | 1.12E-09 |
| rs12038396 | 1 | 156459535 | 1.14E-09 |
| rs17862920 | 2 | 234827995 | 1.14E-09 |
| rs6741751 | 2 | 234827661 | 1.19E-09 |
| rs17862921 | 2 | 234829120 | 1.25E-09 |
| rs2274319 | 1 | 156450873 | 1.42E-09 |
| rs2078371 | 1 | 115677183 | 1.52E-09 |
| rs3790457 | 1 | 156458840 | 1.65E-09 |
| rs840161 | 12 | 57323523 | 1.77E-09 |
| rs7577262 | 2 | 234818869 | 1.97E-09 |
| rs2493212 | 1 | 3084877 | 2.01E-09 |
| rs17863838 | 2 | 234818258 | 2.08E-09 |
| rs17864741 | 2 | 234817840 | 2.24E-09 |
| rs6046121 | 20 | 19455203 | 2.61E-09 |
| rs167769 | 12 | 57503775 | 2.87E-09 |
| rs74766941 | 1 | 3098644 | 2.95E-09 |
| rs11562941 | 2 | 234815167 | 3.02E-09 |
| rs9399930 | 6 | 96854444 | 3.03E-09 |
| rs4814857 | 20 | 19457268 | 3.29E-09 |
| rs6717992 | 2 | 234813712 | 4.10E-09 |
| rs6717978 | 2 | 234813666 | 4.16E-09 |
| rs17864738 | 2 | 234812696 | 4.54E-09 |
| rs10929317 | 2 | 234811911 | 4.88E-09 |
| rs6046142 | 20 | 19476765 | 5.44E-09 |
| rs7558436 | 2 | 234835679 | 7.32E-09 |
| rs12470426 | 2 | 234846401 | 7.56E-09 |
| rs17863841 | 2 | 234837608 | 7.83E-09 |
| rs11562954 | 2 | 234837513 | 8.70E-09 |
| rs2294895 | 20 | 19484894 | 8.77E-09 |
| rs4759272 | 12 | 57438658 | 9.33E-09 |
| rs2143501 | 20 | 19486578 | 1.04E-08 |
| rs4813361 | 20 | 19488952 | 1.07E-08 |
| rs4813360 | 20 | 19488736 | 1.07E-08 |
| rs10807323 | 6 | 12795031 | 1.13E-08 |
| rs8052831 | 16 | 87578039 | 1.15E-08 |
| rs9472790 | 6 | 12879101 | 1.20E-08 |
| rs2294897 | 20 | 19484357 | 1.25E-08 |
| rs9381401 | 6 | 12801967 | 1.36E-08 |
| rs4839826 | 6 | 96853616 | 1.65E-08 |
| rs6924957 | 6 | 96853579 | 1.70E-08 |
| rs7562952 | 2 | 234840357 | 1.74E-08 |
| rs7562971 | 2 | 234840424 | 1.75E-08 |
| rs324011 | 12 | 57502182 | 1.76E-08 |
| rs10490010 | 2 | 234838912 | 1.78E-08 |
| rs76627106 | 20 | 30610164 | 1.82E-08 |
| rs17863842 | 2 | 234842119 | 1.82E-08 |
| rs17868384 | 2 | 234842328 | 1.83E-08 |
| rs67646620 | 2 | 234843225 | 1.85E-08 |
| rs4081947 | 16 | 87579870 | 1.87E-08 |
| rs9381462 | 6 | 12873775 | 1.90E-08 |
| rs10803666 | 2 | 234838947 | 1.92E-08 |
| rs12473889 | 2 | 234844945 | 2.06E-08 |
| rs1571997 | 6 | 12874309 | 2.18E-08 |
| rs12038657 | 1 | 3093302 | 2.25E-08 |
| rs703816 | 12 | 57497005 | 2.35E-08 |
| rs7597116 | 2 | 234832232 | 2.40E-08 |
| rs144017103 | 20 | 30628982 | 2.50E-08 |
| rs7602303 | 2 | 234832233 | 2.93E-08 |
| rs17857135 | 17 | 78262161 | 3.93E-08 |
| rs7300066 | 12 | 4529169 | 3.94E-08 |
| rs13028228 | 2 | 234843309 | 3.99E-08 |
| rs146430923 | 7 | 40373767 | 4.14E-08 |
| rs10774231 | 12 | 4515374 | 4.18E-08 |
| rs17171683 | 7 | 40366619 | 4.35E-08 |
| rs17171687 | 7 | 40369220 | 4.42E-08 |
| rs186166891 | 7 | 40406876 | 4.45E-08 |
| rs17171686 | 7 | 40368926 | 4.54E-08 |
| rs7214019 | 17 | 78235595 | 4.58E-08 |
| rs73322422 | 7 | 40386533 | 4.59E-08 |
| rs4599656 | 6 | 96936682 | 4.68E-08 |
| rs4766241 | 12 | 4523225 | 4.69E-08 |
| rs6489545 | 12 | 4528491 | 4.73E-08 |
| rs6941258 | 6 | 96938856 | 4.74E-08 |
| rs10849061 | 12 | 4523456 | 4.77E-08 |
