# Supplementary material for: A Meta-Analysis of the Genome-Wide Association Studies on Two Genetically Correlated Phenotypes Suggests Four New Risk Loci for Headaches
Source: Phenomics. 2022 Nov 18;3(1):64–76. doi: 10.1007/s43657-022-00078-7 (PMC9883337; doi:10.1007/s43657-022-00078-7)
Supplement: Supplementary file 7 — Supplementary file7 (DOCX 16 KB) [file 43657_2022_78_MOESM7_ESM.docx]

Supplementary table 5: The 4 loci suggested by Meng et al while dropped out in the current study

| Gene | Lead SNP | Chromosome | SNP  position | *p*- migraine | p-headache | p (meta) |
| --- | --- | --- | --- | --- | --- | --- |
| *BTN2A2* | rs2072806 | 6 | 26385093 | 0.50 | 5.30E-09 | 7.51E-08 |
| *IFT81* | rs7300001 | 12 | 110581731 | 0.34 | 8.86E-09 | 1.10E-07 |
| *PTBP2* | rs3748784 | 1 | 97187174 | 0.13 | 1.75E-08 | 1.25E-07 |
| *MACF1* | rs2036465 | 1 | 39575982 | 0.02 | 4.00E-08 | 5.99E-08 |
