# Supplementary material for: A Meta-Analysis of the Genome-Wide Association Studies on Two Genetically Correlated Phenotypes Suggests Four New Risk Loci for Headaches
Source: Phenomics. 2022 Nov 18;3(1):64–76. doi: 10.1007/s43657-022-00078-7 (PMC9883337; doi:10.1007/s43657-022-00078-7)
Supplement: Supplementary file 8 — Supplementary file8 (DOCX 23 KB) [file 43657_2022_78_MOESM8_ESM.docx]

Supplementary table 6: The 14 loci which were not identified by Meng et al (2018) while being significant in the current study.

| Rank | Gene | Lead SNP | Chr | SNP  position | *p* - migraine | *p -*headache | Z-migraine | Z-headache | *p* (meta) |
| --- | --- | --- | --- | --- | --- | --- | --- | --- | --- |
| 13 | *SLC24A3* | rs4814864 | 20 | 19469817 | 2.36x10^-12^ | 8.88x10^-04^ | 7.03 | 3.32 | 2.39x10^-13^ |
| 18 | *SUGCT* | rs77410344 | 7 | 40410924 | 8.45x10^-08^ | 4.84x10^-06^ | 5.39 | 4.57 | 7.44x10^-11^ |
| 19 | *Intergenic (Near JAG1)* | rs6040095 | 20 | 10680221 | 7.98x10^-08^ | 4.67x10^-06^ | 5.38 | 4.58 | 7.78x10^-11^ |
| 20 | *RNF213* | rs12943001 | 17 | 78238645 | 7.93x10^-08^ | 5.37x10^-05^ | -5.36 | -4.04 | 7.35x10^-10^ |
| 21 | *ONECUT2* | rs673939 | 18 | 55153266 | 4.88x10^-05^ | 1.63x10^-07^ | -4.05 | -5.24 | 1.29x10^-09^ |
| 24 | *Intergenic (Near ZCCHC14)* | rs8052831 | 16 | 87578039 | 1.15x10^-08^ | 2.86x10^-03^ | 5.72 | 2.98 | 3.80x10^-09^ |
| 25 | *PLCE1* | rs3891783 | 10 | 96015793 | 3.85x10^-07^ | 7.53x10^-05^ | -5.07 | -3.96 | 4.10x10^-09^ |
| 26 | *CARF* | rs72928613 | 2 | 203839628 | 2.58x10^-05^ | 1.23x10^-06^ | -4.19 | -4.85 | 4.80x10^-09^ |
| 27 | *Intergenic (Near ITPK1)* | rs28540738 | 14 | 93591673 | 3.56x10^-06^ | 9.60x10^-06^ | -4.63 | -4.43 | 4.95x10^-09^ |
| 30 | *MAU2* | rs34858588 | 19 | 19457235 | 3.53x10^-04^ | 3.40x10^-07^ | 3.59 | 5.10 | 1.33x10^-08^ |
| 32 | *Intergenic (Near KCNK17)* | rs72854120 | 6 | 39248533 | 6.22x10^-03^ | 3.99x10^-08^ | -2.68 | -5.49 | 2.81x10^-08^ |
| 33 | *ZNF462* | rs2134063 | 9 | 109695139 | 1.60x10^-03^ | 1.85x10^-07^ | 3.16 | 5.21 | 2.98x10^-08^ |
| 35 | *LOC101927995 (Near TGFBR2)* | rs6791480 | 3 | 30480559 | 7.70x10^-05^ | 4.60x10^-06^ | 3.96 | 4.58 | 3.81x10^-08^ |
| 38 | *Intergenic (Near REST–SPINK2)* | rs781669 | 4 | 57819794 | 4.59x10^-03^ | 1.09x10^-07^ | 2.83 | 5.31 | 4.67x10^-08^ |

Chr: chromosome

The Z values (ratio of effect size to standard error) stand for the specific SNP effect contribution from each cohort.
