# Supplementary material for: A Meta-Analysis of the Genome-Wide Association Studies on Two Genetically Correlated Phenotypes Suggests Four New Risk Loci for Headaches
Source: Phenomics. 2022 Nov 18;3(1):64–76. doi: 10.1007/s43657-022-00078-7 (PMC9883337; doi:10.1007/s43657-022-00078-7)
Supplement: Supplementary file 9 — Supplementary file9 (DOCX 26 KB) [file 43657_2022_78_MOESM9_ESM.docx]

| Rank | Gene | Lead SNP | Chr | SNP  position | Reported by Gormley et al | Reported by Meng et al | Reported by 23andMe |
| --- | --- | --- | --- | --- | --- | --- | --- |
| 1 | *LRP1-STAT6-SDR9C7* | rs11172113 | 12 | 57527283 | Yes | Yes | Yes |
| 2 | *FHL5-UFL1* | rs9486715 | 6 | 97059769 | Yes | Yes | Yes |
| 3 | *PHACTR1* | rs9349379 | 6 | 12903957 | Yes | Yes | Yes |
| 4 | *TRPM8-HJURP* | rs2362290 | 2 | 234825369 | Yes | Yes | Yes |
| 5 | *PRDM16* | rs10218452 | 1 | 3075597 | Yes | Yes | Yes |
| 6 | *MEF2D* | rs2282286 | 1 | 156452870 | Yes | Yes | Yes |
| 7 | *Intergenic (Near TSPAN2–NGF)* | rs12134493 | 1 | 115677946 | Yes | Yes | Yes |
| 8 | *Intergenic (Near ADAMTSL4–ECM1)* | rs6693567 | 1 | 150510660 | Yes | Yes | No |
| 9 | *Intergenic (Near GPR149)* | rs34097149 | 3 | 154263175 | Yes | Yes | No |
| 10 | *Intergenic (Near FGF6)* | rs10774231 | 12 | 4515374 | Yes | Yes | Yes |
| 11 | *LINC02210-CRHR1-MAPT* | rs117368197 | 17 | 43715924 | No | Yes | No |
| 12 | *ASTN2* | rs10759844 | 9 | 119249326 | Yes | Yes | No |
| 13 | *SLC24A3* | rs4814864 | 20 | 19469817 | Yes | No | Yes |
| 14 | *CFDP1* | rs11149826 | 16 | 75435140 | Yes | Yes | No |
| 15 | *PLEKHA1 (ARMS2–HTRA1)* | rs78438709 | 10 | 124201071 | Yes | Yes | No |
| 16 | *MRVI1* | rs4909945 | 11 | 10673739 | Yes | Yes | No |
| 17 | *Intergenic (Near GJA1)* | rs9490318 | 6 | 121860207 | Yes | Yes | No |
| 18 | *SUGCT* | rs77410344 | 7 | 40410924 | Yes | No | Yes |
| 19 | *Intergenic (Near JAG1)* | rs6040095 | 20 | 10680221 | Yes | No | No |
| 20 | *RNF213* | rs12943001 | 17 | 78238645 | Yes | No | Yes |
| 21 | *ONECUT2* | rs673939 | 18 | 55153266 | No | No | No |
| 22 | *NOL4L* | rs159058 | 20 | 31108108 | No | Yes | No |
| 23 | *Intergenic (Near ZCCHC2)* | rs4941139 | 18 | 60162791 | No | Yes | No |
| 24 | *Intergenic (Near ZCCHC14)* | rs8052831 | 16 | 87578039 | Yes | No | Yes |
| 25 | *PLCE1* | rs3891783 | 10 | 96015793 | Yes | No | No |
| 26 | *CARF* | rs72928613 | 2 | 203839628 | Yes | No | No |
| 27 | *Intergenic (Near ITPK1)* | rs28540738 | 14 | 93591673 | Yes | No | No |
| 28 | *CHRM4* | rs2067482 | 11 | 46406767 | No | Yes | No |
| 29 | *CAMK1D* | rs10752269 | 10 | 12692902 | No | Yes | No |
| 30 | *MAU2* | rs34858588 | 19 | 19457235 | No | No | No |
| 31 | *MYO1H* | rs6606710 | 12 | 109848903 | No | Yes | No |
| 32 | *Intergenic (Near KCNK17)* | rs72854120 | 6 | 39248533 | No | No | No |
| 33 | *ZNF462* | rs2134063 | 9 | 109695139 | No | No | No |
| 34 | *Intergenic (Near CDKN2C)* | rs7555006 | 1 | 51480258 | No | Yes | No |
| 35 | *LOC101927995 (Near TGFBR2)* | rs6791480 | 3 | 30480559 | Yes | No | No |
| 36 | *TJP2* | rs7850547 | 9 | 71747208 | No | Yes | No |
| 37 | *NUFIP2* | rs8614 | 17 | 27588806 | No | Yes | No |
| 38 | *Intergenic (Near REST–SPINK2)* | rs781669 | 4 | 57819794 | Yes | No | No |

Chr: chromosome

Supplementary Table 7: Loci comparison among the 4 studies (the current study, Gormley et al, Meng et al and 23andMe results)
